# Associations between classic psychedelics and nicotine dependence in a nationally representative sample

**DOI:** 10.1038/s41598-022-14809-3

**Published:** 2022-06-22

**Authors:** Grant Jones, Joshua Lipson, Matthew K. Nock

**Affiliations:** 1 Department of Psychology, Harvard University, 33 Kirkland St, Cambridge, MA 02138 USA; 2 Teacher’s College, Columbia University, New York, USA

**Keywords:** Psychology, Human behaviour

## Abstract

Tobacco use is the single largest cause of preventable death worldwide, but none of the established treatments aimed at smoking cessation work for a majority of smokers. As such, there is an urgent need for interventions capable of reliably treating nicotine addiction. The use of classic psychedelics has been associated with lower odds of many forms of substance dependence. Here we tested whether lifetime use of classic psychedelics (tryptamine, lysergamide, and phenethylamine) is associated with lower odds of current nicotine dependence. We tested these associations in a sample of 214,505 adult participants in the National Survey on Drug Use and Health (2015–2019) using multivariable logistic regression models. Lifetime psilocybin use was associated with reduced odds of odds of current nicotine dependence (aOR 0.87–0.93). Lifetime use of peyote and mescaline also conferred reduced odds of multiple subdomains of a main nicotine dependence measure (Nicotine Dependence Syndrome Scale [NDSS]) (aOR 0.79–0.91). Conversely, lifetime use of LSD was associated with increased odds of nicotine dependence (aOR 1.17–1.24). Psilocybin, mescaline, and peyote use are associated with lowered odds of nicotine dependence. Experimental studies are needed to establish whether these associations are causal. These results make the case for further research into the efficacy of both tryptamine and phenethylamine psychedelics in promoting smoking cessation.

## Introduction

Tobacco is the single largest cause of preventable death worldwide, killing 8.7 million people each year, a number that continues to increase, despite the steady expansion of prevention measures across the globe^1^. In addition, tens of millions of individuals suffer from a range of avoidable respiratory, cardiovascular, and other illnesses as a result of smoking tobacco, and over the past two years, studies have demonstrated that cigarette smokers are more likely to be hospitalized or die from COVID-19^1^. In the United States, 70% of current adult smokers say that they want to quit, and in 2018, 55% reported attempting to quit at least once in the past year^2,3^. However, to date, even the most effective pharmacological and behavioral smoking cessation treatments fail to promote long-term abstinence in a large majority of people who use them^4,5^. The development of more effective, reliable, and long-lasting smoking cessation treatments represents one of the most important health needs around the world^1^.

In recent years, researchers have begun to explore the use of classic psychedelics (Greek for “mind-manifesting”), typically in the context of a psychotherapeutic framework, as a treatment for a host of different forms of addiction and substance abuse, including addiction to smoking tobacco. Classic psychedelics are found in nature or can be synthesized from natural compounds, and have been demonstrated to elicit mystical-type experiences characterized by self-transcendent awe that can have lasting personal and spiritual significance^6–9^. On a pharmacological level, they are defined by their agonist activity at the 5-HT2A receptor site^10^. Six of the main substances included in this class are: the tryptamine psilocybin (the active component within “magic mushrooms”), the phenethylamines peyote (a psychoactive cactus) and mescaline (the active compound within peyote), ayahuasca (a psychoactive brew including Banisteriopsis caapi and Psychotria viridis), DMT (the active component within ayahuasca), and lysergic acid diethylamide (LSD; synthesized from the ergot fungus).

Recent evidence has linked classic psychedelics to lowered odds of substance abuse and dependence. In a sample of 44,678 respondents who reported lifetime use of illicit opioids in the National Survey on Drug Use and Health (NSDUH), Pisano et al. (2017) found that psychedelic use was associated with 27% reduced risk of past-year opioid dependence, and 40% reduced risk of past-year opioid abuse^11^. Smaller-scale attempts at measuring the association between naturalistic use of classic psychedelics, on one hand, and misuse of other addictive substances, including alcohol, cannabis, and stimulants, on the other, have yielded results suggesting an association between psychedelic use and large-scale remission from abuse disorders^12,13^. In addition, recent evidence has emerged to suggest that the naturalistic use of mescaline, specifically, is associated with reductions in the symptoms of alcohol and drug use disorders^14^.

Additionally, over the past decade, a line of research by Johnson and colleagues has suggested an association between the use of classic psychedelics and smoking cessation in both experimental and naturalistic settings. First, in an open-label pilot study that combined two or three doses of psilocybin with a program of cognitive behavioral therapy (CBT) targeted at smoking cessation, Johnson et al. (2014) found that 12 of 15 (80%) participants met the criteria for smoking abstinence at 6-month follow-up^15^. In a follow-up study on psilocybin-assisted smoking cessation therapy, Johnson et al. (2017a) identified a 67% rate of smoking abstinence at 12-month follow-up, compared to a 31% rate of abstinence at 12 months post-treatment for gold standard smoking cessation medications^16^. However, these researchers note their findings are limited by the small size and homogeneity of their sample.

Johnson et al. (2017b) complemented these experimental clinical data by examining the effects of psychedelics on smoking behavior in a naturalistic context^17^. The authors recruited 358 individuals who reported having quit or reduced smoking after ingesting any psychedelic in a non-laboratory setting a year or more ago, and found that at the time of survey, 38% of participants reported continuous smoking cessation, and an additional 28% reported persistent reduction from pre-psychedelic smoking levels.

Despite the novel contribution Johnson et al. (2017b) makes to the literature, there are significant limitations to this study as well. The authors acknowledge the issue of selection bias, given that the survey was distributed to websites visited by individuals interested in psychedelics (e.g., www.erowid.org, www.shroomery.org). Additionally, the sample was considerably male-skewed, with only 15% of respondents identifying as female. Their sample also was heavily skewed toward users of LSD and psilocybin, with only a small minority reporting use of phenethylamine psychedelics.

The current study seeks to address the limitations of past studies by testing the associations between lifetime use of classic psychedelics and current (past month) nicotine dependence using a large-scale nationally-representative sample. Although such an approach cannot be used to infer a causal association between psychedelic use and nicotine dependence, the large and representative nature of this sample allows us to glean critical insights about the robustness of the associations between psychedelic use and nicotine dependence and to build on the existing small-scale research on this matter.

## Method

### Sample

Data are from the National Survey on Drug Use and Health (NSDUH; 2015–2019) (unweighted *N* = 214,505), a survey that assesses behavior, health, and substance use outcomes in U.S citizens ages 12 years and older. Currently incarcerated individuals, individuals experiencing homelessness, and active-duty military service members are not included in the NSDUH. Given that data for this project are publicly available, this study was determined to be exempt from review by the Harvard IRB. All study procedures were carried out in accordance with the relevant guidelines and regulations.

### Measures

#### Dependent variables

Our main dependent variables were two measures assessing past month nicotine dependence within the NSDUH: composite scores on the Nicotine Dependence Syndrome Scale (NDSS) and the Fagerstrom Test of Nicotine Dependence (FTND), recently renamed and known elsewhere as the Fagerstrom Test of Cigarette Dependence (FTCD). The NSDUH NDSS composite measure is based on 17 questions that assess various aspects of nicotine dependence. Scores on each question ranged from 1 (not at all true) to 5 (extremely true) (some items were reverse coded). The NDSS composite score represents the mean of these 17 questions. Individuals were coded as past–month nicotine–dependent based on the NDSS if they received a score of 2.75 or above on the composite NDSS measure. The NSDUH FTND measure, by contrast, is based on a single criterion: whether one smokes within 30 min of waking up. Individuals meeting this criterion were coded as dependent based on the FTND.

In addition to estimating models based on these two composite scores, we also estimated models based on subdomains of the NDSS. The NSDUH indicates that the 17 questions comprising the NDSS can be grouped into five subdomains: smoking drive (compulsion to smoke), nicotine tolerance, continuous smoking, behavioral priority (prioritizing smoking over other reinforcing activities), and stereotypy (having fixed smoking patterns). Thus, we took the mean for all questions within each subdomain and rounded these values to the nearest integer to create ordinal subdomain variables ranging from 1 (low dependence) to 5 (high dependence).

#### Independent variables/covariates

Our main independent variables were NSDUH items assessing lifetime use (yes/no) of the following classic psychedelics: psilocybin, peyote, mescaline, and LSD. Additionally, to test the specificity of any observed associations, we included as independent variables items assessing lifetime use of various legal/medicinal and illegal substances: MDMA/ecstasy, heroin, PCP, inhalants, cocaine, pain relievers, tranquilizers, stimulants, sedatives, and marijuana. Several sociodemographic variables were included as a priori covariates: sex, age, race/ethnicity, marital status, income, educational attainment, and self-reported engagement in risky behavior.

### Analysis plan

We used multivariable logistic regression to assess the associations between each independent variable and the two main nicotine dependence measures (the NDSS and FTND composite variables). For our first and second models, we simultaneously entered all of our predictor variables, with the aim of gauging their respective associations with the NDSS and the FTND composites, respectively.

Additionally, given that our subdomain variables are scored ordinally, we ran five ordinal logistic regression models to test the associations between our predictor variables and each of the five nicotine dependence subdomains of the NDSS. For each of these models, we simultaneously entered all of our predictor variables, and examined their association with the given NDSS subdomain.

We used the ‘Survey’ package in R to incorporate the complex survey design and sampling weights of the NSDUH into our models^18^. Additionally, we used the ‘lmtest’ package to conduct our significance tests for the ordinal logistic regression models in this study^19^, as the ‘Survey’ package does not generate p-values for this class of models.

## Results

Demographic differences between those who do versus do not meet criteria for past-month nicotine dependence are presented in Table 1. Individuals with nicotine dependence were more likely to be divorced or never married, less formally educated, younger, Non-Hispanic White, lower-income, and to report slightly more engagement in risky-behavior.

**Table 1:** Demographic characteristics of those with and without nicotine dependence (ND).

Characteristic	Does not have ND (weighted %) (N = 188,367) (%)	Has ND (weighted %) (N = 26,138) (%)	p-value^1^
**Marital status**			< 0.001
Married	54	36	
Widowed	6.0	5.5	
Divorced or separated	13	24	
Never been married	28	35	
**Education**			< 0.001
5th grade or lower	1.3	0.5	
6th grade	1.2	0.4	
7th grade	0.4	0.7	
8th grade	1.0	1.9	
9th grade	1.5	3.6	
10th grade	1.7	5.0	
11th or 12th grade	4.5	9.0	
High school diploma/GED	23	37	
Some college credit	21	24	
Associate's	9.4	8.2	
Bachelor's or higher	34	9.9	
**Age**			< 0.001
18–25	14	12	
26–34	16	19	
35–49	24	29	
50+	46	40	
**Sex**			< 0.001
Male	48	53	
Female	52	47	
**Race**			< 0.001
Non-Hispanic White	63	74	
Non-Hispanic Black	12	13	
Non-Hispanic Native American/Alaska Native	0.5	0.9	
Non-Hispanic Native Hawaiian/Pacific Islander	0.4	0.4	
Non-Hispanic Asian	6.1	1.7	
Non-Hispanic more than one race	1.6	2.7	
Hispanic	17	8.0	
**Yearly household income**			< 0.001
< $20,000	15	29	
$20,000–$49,999	29	36	
$50,000–$74,999	16	14	
$75,000 +	40	21	
**Self-reported engagement in risky behavior**			< 0.001
Never	56	48	
Seldom	32	33	
Sometimes	11	16	
Always	1.2	2.8	

*ND* Nicotine Dependence.

^1^Chi-squared test with Rao & Scott's second-order correction.

Results from the logistic regression models testing the associations between lifetime use of all substances and the NDSS and FTND composite measures are presented in Table 2. As hypothesized, lifetime psilocybin use was associated with lower odds of current (past month) nicotine dependence as measured by both the NDSS (aOR 0.90) and FTND (aOR 0.87). No other substances examined were associated with lower odds of these measures. Several, including LSD, were associated with increased odds of nicotine dependence.

**Table 2:** Associations between lifetime substance use and two measures of nicotine dependence.

Lifetime use	Nicotine dependence (NDSS)		Nicotine dependence (FTND)	
	aOR (95% CI)^1^	p-value	aOR (95% CI)	p-value
**Psilocybin**	0.90* (0.83, 0.98)	**0.017**	0.87* (0.78, 0.97)	**0.021**
Peyote	0.92 (0.77, 1.10)	0.310	0.99 (0.85, 1.16)	0.941
Mescaline	0.90 (0.75, 1.06)	0.178	0.88 (0.76, 1.02)	0.075
LSD	1.22*** (1.13, 1.32)	2.37e−04	1.17** (1.07, 1.28)	0.004
MDMA/ecstasy	1.13* (1.03, 1.24)	0.018	1.13* (1.03, 1.25)	0.017
PCP	1.17* (1.02, 1.34)	0.026	1.06 (0.93, 1.21)	0.316
Cocaine	1.56*** (1.47, 1.67)	6.41e−08	1.64*** (1.51, 1.78)	1.97e−07
Heroin	1.83*** (1.62, 2.07)	1.48e−06	1.89*** (1.69, 2.11)	4.20e−07
Inhalants	1.08 (0.99, 1.17)	0.064	1.00 (0.91, 1.10)	0.971
Pain relievers	1.28*** (1.19, 1.37)	2.74e−05	1.13*** (1.07, 1.19)	9.61e−04
Tranquilizers	1.20*** (1.13, 1.27)	6.83e−05	1.18*** (1.11, 1.26)	2.07e−04
Stimulants	0.99 (0.93, 1.06)	0.799	0.95 (0.88, 1.02)	0.148
Sedatives	1.10* (1.02, 1.19)	0.015	1.03 (0.95, 1.11)	0.397
Marijuana	3.03*** (2.80, 3.28)	1.44e−10	2.71*** (2.53, 2.90)	1.00e−10

Significant values conferring lowered odds of nicotine dependence are in bold.

^1^*p < 0.05; **p < 0.01; ***p < 0.001; *aOR* adjusted odds ratio, *CI* confidence interval.

Results from the models testing the associations between lifetime substance use and the five subdomains of the NDSS are presented in Table 3. Mescaline use was associated with lower odds of all five subdomains of nicotine dependence (aOR 0.79–0.86) and peyote use was associated with lowered odds of the nicotine tolerance (aOR 0.89) and continuous smoking (aOR 0.88) subdomains. Psilocybin use was associated with lower odds of the behavioral priority subdomain (aOR 0.93). Aside from one instance of sedatives conferring decreased odds of the stereotypy subdomain (aOR 0.93), all other substances were either not associated with nicotine dependence or associated with increased odds of nicotine dependence. Additionally, each of the aforementioned demographic covariates shared significant, varying relationships to nicotine dependence across all of our models.

**Table 3:** Associations between lifetime substance use and the five NDSS subdomains.

Lifetime use	Smoking drive		Nicotine tolerance		Continuous smoking		Behavioral priority		Stereotypy	
	aOR (95% CI)^1^	p-value	aOR (95% CI)	p-value	aOR (95% CI)	p-value	aOR (95% CI)	p-value	aOR (95% CI)	p-value
Psilocybin	0.96 (0.90, 1.01)	0.170	0.96 (0.91, 1.02)	0.188	0.98 (0.92, 1.04)	0.475	0.93* (0.88, 0.99)	**0.048**	0.97 (0.91, 1.03)	0.335
Peyote	0.90 (0.81, 0.99)	0.061	0.89* (0.81, 0.98)	**0.038**	0.88* (0.80, 0.96)	**0.022**	0.91 (0.83, 1.00)	0.081	0.90 (0.82, 0.99)	0.058
Mescaline	0.82** (0.73, 0.92)	**0.008**	0.79** (0.72, 0.88)	**0.002**	0.84* (0.75, 0.93)	**0.010**	0.83** (0.74, 0.92)	**0.008**	0.86* (0.77, 0.95)	**0.015**
LSD	1.24*** (1.17, 1.32)	4.54e−05	1.21*** (1.14, 1.28)	1.61e−04	1.22*** (1.15, 1.29)	8.64e−05	1.21*** (1.14, 1.28)	1.09e−04	1.17*** (1.11, 1.24)	3.55e−04
MDMA/ecstasy	1.25*** (1.19, 1.32)	1.49e−05	1.32*** (1.25, 1.38)	1.57e−06	1.26*** (1.19, 1.33)	1.59e−05	1.23*** (1.17, 1.30)	1.90e−05	1.26*** (1.20, 1.31)	4.01e−06
PCP	1.01 (0.92, 1.12)	0.795	1.00 (0.91, 1.10)	0.954	1.01 (0.92, 1.11)	0.851	1.03 (0.94, 1.13)	0.521	1.03 (0.93, 1.13)	0.597
Cocaine	1.70*** (1.62, 1.79)	5.24e−09	1.68*** (1.60, 1.76)	3.50e−09	1.66*** (1.58, 1.74)	7.14e−09	1.67*** (1.60, 1.75)	3.61e−09	1.60*** (1.53, 1.68)	9.75e−09
Heroin	1.73*** (1.56, 1.93)	3.22e−06	1.70*** (1.54, 1.88)	2.72e−06	1.57*** (1.42, 1.73)	9.72e−06	1.51*** (1.36, 1.67)	2.38e−05	1.31*** (1.19, 1.44)	3.81e−04
Inhalants	1.03 (0.98, 1.10)	0.285	1.02 (0.96, 1.07)	0.599	1.00 (0.94, 1.06)	0.960	1.01 (0.96, 1.07)	0.643	0.96 (0.90, 1.02)	0.190
Pain Relievers	1.12*** (1.07, 1.16)	4.69e−04	1.11*** (1.07, 1.16)	7.16e−04	1.10** (1.06, 1.15)	0.002	1.09** (1.05, 1.14)	0.003	1.06* (1.02, 1.10)	0.024
Tranquilizers	1.17*** (1.12, 1.22)	5.98e−05	1.17*** (1.12, 1.21)	4.09e−05	1.14*** (1.09, 1.19)	2.58e−04	1.15*** (1.10, 1.21)	1.47e−04	1.10** (1.05, 1.15)	0.003
Stimulants	1.10*** (1.06, 1.14)	8.40e−04	1.11*** (1.07, 1.16)	5.11e−04	1.09** (1.05, 1.13)	0.002	1.10** (1.06, 1.14)	0.001	1.09** (1.05, 1.13)	0.002
Sedatives	1.00 (0.95, 1.05)	0.966	0.98 (0.94, 1.03)	0.427	0.98 (0.93, 1.02)	0.337	0.96 (0.92, 1.01)	0.163	0.93* (0.89, 0.97)	0.013
Marijuana	2.80*** (2.69, 2.91)	2.66e−12	2.80*** (2.69, 2.92)	2.45e−12	2.79*** (2.68, 2.90)	1.93e−12	2.78*** (2.66, 2.90)	3.53e−12	2.68*** (2.57, 2.79)	4.39e−12

Significant values conferring lowered odds of nicotine dependence are in bold.

^1^*p < 0.05; **p < 0.01; ***p < 0.001, *aOR* adjusted odds ratio, *CI* confidence interval.

## Discussion

The primary finding from this study is that the lifetime use of classic psychedelics (both tryptamine and phenethylamine) was associated with lower odds of current nicotine dependence as well as the sub-domains underlying this construct. Psilocybin conferred lowered odds of both composite measures of past month nicotine dependence in the NSDUH (the NDSS and the FTND), and peyote and mescaline conferred lowered odds of various subdomains of the NDSS. Aside from a singular instance of sedatives conferring lowered odds of one nicotine dependence subdomain, all other substances either shared no association with nicotine dependence or conferred increased odds of nicotine dependent behavior.

Although these results cannot be used to infer a causal association, they document the robust association between the lifetime use of psilocybin, peyote, and mescaline and the lowered odds of nicotine dependence. Moreover, these results suggest the possibility that psychedelic use might also be protective against future nicotine dependence, beyond the single time point captured in the dataset we analyzed. These results are timely, as the National Institute of Health (NIH) recently funded a multi-site clinical trial testing psilocybin for treating nicotine dependence^20^. That trial marks the first time in 50 years that the NIH is funding a trial involving psychedelics. This study further supports the need for trials that test the therapeutic efficacy of psilocybin, peyote, and mescaline for treating nicotine dependence and potentially other substance use disorders as well.

These findings also are consistent with other population-based survey research on classic psychedelics suggesting that naturalistic use of LSD in particular is associated with increased odds of adverse outcomes^21,22^, despite evidence for LSD’s therapeutic efficacy under clinical administration^23^. Negative outcomes have been associated with the use of some psychedelics, such as paranoia, stress, anxiety, and increased odds of psychosis^24^, and might underlie the association between LSD use and increased odds of nicotine dependence. Additionally, it is possible that LSD use in particular is confounded with other factors associated with nicotine dependence. Alternatively, naturalistic use of LSD might be associated with different cultural framing and settings than naturalistic use of psilocybin, accounting for a higher probability of adverse outcomes. Further investigation into the potentially adverse effects of classic psychedelics, and the potential harm of LSD use in naturalistic contexts, is warranted.

### Limitations

These results should be interpreted in the context of several major limitations. First, these results are based on cross-sectional data and cannot be used to draw causal inferences about the observed associations. Third-variable factors and pre-existing differences between those who have versus have not used psilocybin, peyote, and mescaline very possibly contribute to the associations we observed. Longitudinal studies and clinical trials are needed to better understand the temporal and causal associations between psilocybin, peyote, and mescaline use and lowered odds of nicotine dependence. Additional investigations into potential third variable factors can have the additional benefit of identifying factors that may be protective against nicotine dependence as well.

Second, the questions in the NSDUH are based on self-report, and thus, for substance use and nicotine dependence questions, under-reporting may be a complicating factor in our analyses and conclusions. Future studies that strictly monitor and observe substance use can address this limitation.

Third, this study cannot clearly establish that classic psychedelic use took place prior to the onset of nicotine dependence, given the overlapping time horizons of these two measures. However, classic psychedelic use was assessed over an entire lifetime and nicotine dependence was assessed over the past month; hence, many individuals likely engaged in use of classic psychedelics prior to the onset of nicotine dependence.

Fourth, we cannot establish recency or frequency of classic psychedelic use in our study, given the binary nature of our lifetime use variable. Nevertheless, causal interpretations of our findings remain plausible despite this limitation, as psilocybin has been shown to elicit lasting reductions in nicotine dependence after just two to three administrations^16^.

Fifth, multicollinearity represents a possible limitation to our findings. Multicollinearity refers to substantial levels of correlation between independent variables; furthermore, multicollinearity increases the standard errors within a given model and can thus result in unstable model estimates. Multicollinearity is primarily measured by Variance Inflation Factors (VIFs) and a VIF above 10 indicates the presence of multicollinearity for a given independent variable. We report the VIFs for the independent variables within our study in Supplemental Table 1. As indicated within this table, some of our independent variables have VIFs above 10, indicating that multicollinearity is present within our study. However, the impact of multicollinearity on our results is likely limited, as our large sample size serves to minimize the impact that inflated standard errors may otherwise have on the integrity of our models^25^. Nevertheless, future studies can further address this limitation by using modeling approaches such as ridge regression that are designed to handle multicollinearity.

Lastly, our approach to calculating p-values in this study features limitations as well. As previously indicated, we used the ‘lmtest’ package to conduct Wald tests (quasi-t tests) and generate p-values for our ordinal regression models^19^. However, one can plausibly conduct z-tests for the ordinal regression models within this study as well. Z-tests are typically used when one is certain that their data are normally distributed, but can also be used for a sample with an unknown distribution if the sample size is large, as is the case with our study^26^. For transparency, we also report the p-values yielded from z-tests for our ordinal regression models in Supplemental Table 2. As one can see from this table, our pattern of results remain largely unchanged, regardless of the approach one takes for significance testing within this study.

### Potential mechanisms

Several distinct pharmacological and psychological mechanisms may underlie our observed findings, if the associations reported above are indeed causal. The effects of psilocybin, peyote, and mescaline on the serotonin system may mediate the link between these substances and lowered odds of nicotine dependence. It is currently understood that these substances primarily act as serotonin (5-HT2A) receptor agonists, binding to a site typically targeted by serotonin. It has been suggested that downstream changes in functional connectivity, information processing, and neuroplasticity lead to psychedelic states of consciousness with therapeutic potential^27–29^. Aberrant serotonin neurotransmission is implicated in many elements of addiction, including attentional and motivational biases and impulse control deficits associated with compulsive substance use^30–33^. Accordingly, serotonergic pharmacological interventions have been proposed as a promising treatment option for nicotine dependence as well^34,35^. Future research that deepens our knowledge of the serotonergic effects of classic psychedelics may shed light on their potential role in treating nicotine addiction.

In addition, the spiritual experiences elicited by classic psychedelics, best characterized as experiences of self-transcendent awe, represent a plausible core psychological mechanism mediating our associations. In the open label trial of psilocybin for nicotine dependence conducted by Johnson et al. (2014), reductions in nicotine dependence were significantly correlated with measures of mystical experience on psilocybin session days, as well as with retrospective ratings of the personal meaning and spiritual significance of the psilocybin experience^36^. Spirituality is often linked with positive substance abuse recovery outcomes, more broadly^37–39^. Additional inquiries into the role of self-transcendent spiritual experiences in the alleviation of addiction can shed light on the potential link between classic psychedelics and lowered odds of nicotine dependence.

There are several other potential psychological mechanisms that may underlie our observed relationships as well, many of which have been described by Bogenschutz and Pommy^30^. Classic psychedelics can cause lasting improvements in mood and well-being^6^ that in turn may reduce the risk for addiction and relapse^40,41^. Psychedelics also may cause shifts in personality traits that lead to downstream reductions in nicotine dependence as well^42–44^. The aforementioned changes elicited by psychedelic experiences (e.g. increases in mood and spirituality, changes in personality) may also elicit increases in self-efficacy and motivation that further support reductions in addictive behavior^30,45^. Importantly, these proposed mechanisms are preliminary, and additional research is needed to help determine which factors mediate the link between use of classic psychedelics and lowered odds of nicotine dependence.

## Conclusion

This study provides preliminary evidence supporting the association between use of classic psychedelics of both the tryptamine and phenethylamine classes, and lowered odds of nicotine dependence. Future clinical trials are needed to test whether this link is causal. Additional inquiry may also identify mediators and third-variable factors underlying our findings, which may act as protective factors against nicotine dependence. This study represents an important step toward understanding the link between use of classic psychedelics and smoking behavior in a naturalistic, large-sample context, as well as incremental progress towards reducing rates of nicotine addiction and stemming a significant source of morbidity and mortality worldwide.

## Supplementary Information

Supplementary Tables.

## Data Availability

The data supporting the findings from this project are publicly available at the Substance Abuse & Mental Health Data Archive (SAMHDA) at the following web address: https://www.datafiles.samhsa.gov/.
