# Supplementary material for: Associations between classic psychedelics and nicotine dependence in a nationally representative sample
Source: Sci Rep. 2022 Jun 22;12:10578. doi: 10.1038/s41598-022-14809-3 (PMC9216303; doi:10.1038/s41598-022-14809-3)
Supplement: Supplementary file 1 — Supplementary Tables. [file 41598_2022_14809_MOESM1_ESM.docx]

**Supplemental Table 1.** Variance Inflation Factors (VIFs) for lifetime classic psychedelic use (Psilocybin, Peyote, Mescaline, LSD) across all seven nicotine dependence models

| Lifetime Use | Nicotine Dependence VIFs (NDSS) | Nicotine Dependence VIFs (FTND) | Smoking Drive VIFs | Nicotine Tolerance VIFs | Continuous Smoking VIFs | Behavioral Priority VIFs | Stereotypy VIFs |
| --- | --- | --- | --- | --- | --- | --- | --- |
| Psilocybin | 12.8 | 26.7 | 9.6 | 11.3 | 12.0 | 9.6 | 14.3 |
| Peyote | 4.4 | 7.1 | 9.8 | 12.2 | 5.7 | 7.2 | 5.9 |
| Mescaline | 4.9 | 6.8 | 6.5 | 5.3 | 7.7 | 7.7 | 8.0 |
| LSD | 9.1 | 26.2 | 9.8 | 8.9 | 8.2 | 7.5 | 6.4 |

**Supplemental Table 2.** Associations between lifetime substance use and the five NDSS subdomains (including p-values yielded from Z-Tests)

|  | Smoking Drive | | Nicotine Tolerance | | Continuous Smoking | | Behavioral Priority | | Stereotypy | |
| --- | --- | --- | --- | --- | --- | --- | --- | --- | --- | --- |
| Lifetime Use | aOR (95% CI)^1^ | p-value | aOR (95% CI) | p-value | aOR (95% CI) | p-value | aOR (95% CI) | p-value | aOR (95% CI) | p-value |
| Psilocybin | 0.96 (0.90, 1.01) | 0.136 | 0.96 (0.91, 1.02) | 0.154 | 0.98 (0.92, 1.04) | 0.456 | **0.93* (0.88, 0.99)** | **0.022** | 0.97 (0.91, 1.03) | 0.309 |
| Peyote | **0.90* (0.81, 0.99)** | **0.032** | **0.89* (0.81, 0.98)** | **0.015** | **0.88** (0.80, 0.96)** | **0.006** | **0.91* (0.83, 1.00)** | **0.049** | **0.90* (0.82, 0.99)** | **0.030** |
| Mescaline | **0.82*** (0.73, 0.92)** | **6.80e-04** | **0.79*** (0.72, 0.88)** | **1.11e-05** | **0.84** (0.75, 0.93)** | **0.001** | **0.83*** (0.74, 0.92)** | **6.77e-04** | **0.86** (0.77, 0.95)** | **0.003** |
| LSD | 1.24*** (1.17, 1.32) | 2.77e-13 | 1.21*** (1.14, 1.28) | 6.15e-10 | 1.22*** (1.15, 1.29) | 1.80e-11 | 1.21*** (1.14, 1.28) | 7.09e-11 | 1.17*** (1.11, 1.24) | 2.80e-08 |
| MDMA/Ecstasy | 1.25*** (1.19, 1.32) | 4.35e-17 | 1.32*** (1.25, 1.38) | 2.66e-28 | 1.26*** (1.19, 1.33) | 7.73e-17 | 1.23*** (1.17, 1.30) | 3.56e-16 | 1.26*** (1.20, 1.31) | 5.98e-23 |
| PCP | 1.01 (0.92, 1.12) | 0.789 | 1.00 (0.91, 1.10) | 0.952 | 1.01 (0.92, 1.11) | 0.846 | 1.03 (0.94, 1.13) | 0.505 | 1.03 (0.93, 1.13) | 0.584 |
| Cocaine | 1.70*** (1.62, 1.79) | 1.53e-100 | 1.68*** (1.60, 1.76) | 5.83e-110 | 1.66*** (1.58, 1.74) | 7.61e-94 | 1.67*** (1.60, 1.75) | 3.47e-109 | 1.60*** (1.53, 1.68) | 1.35e-87 |
| Heroin | 1.73*** (1.56, 1.93) | 4.24e-24 | 1.70*** (1.54, 1.88) | 4.88e-25 | 1.57*** (1.42, 1.73) | 8.14e-19 | 1.51*** (1.36, 1.67) | 2.26e-15 | 1.31*** (1.19, 1.44) | 3.85e-08 |
| Inhalants | 1.03 (0.98, 1.10) | 0.256 | 1.02 (0.96, 1.07) | 0.586 | 1.00 (0.94, 1.06) | 0.958 | 1.01 (0.96, 1.07) | 0.631 | 0.96 (0.90, 1.02) | 0.157 |
| Pain Relievers | 1.12*** (1.07, 1.16) | 9.37e-08 | 1.11*** (1.07, 1.16) | 5.07e-07 | 1.10*** (1.06, 1.15) | 7.10e-06 | 1.09*** (1.05, 1.14) | 6.87e-05 | 1.06** (1.02, 1.10) | 0.007 |
| Tranquilizers | 1.17*** (1.12, 1.22) | 1.78e-12 | 1.17*** (1.12, 1.21) | 1.32e-13 | 1.14*** (1.09, 1.19) | 6.42e-09 | 1.15*** (1.10, 1.21) | 3.69e-10 | 1.10*** (1.05, 1.15) | 4.78e-05 |
| Stimulants | 1.10*** (1.06, 1.14) | 9.28e-07 | 1.11*** (1.07, 1.16) | 1.33e-07 | 1.09*** (1.05, 1.13) | 2.68e-05 | 1.10*** (1.06, 1.14) | 3.18e-06 | 1.09*** (1.05, 1.13) | 2.25e-05 |
| Sedatives | 1.00 (0.95, 1.05) | 0.965 | 0.98 (0.94, 1.03) | 0.405 | 0.98 (0.93, 1.02) | 0.310 | 0.96 (0.92, 1.01) | 0.129 | **0.93** (0.89, 0.97)** | **0.002** |
| Marijuana | 2.80*** (2.69, 2.91) | 0.00e+00 | 2.80*** (2.69, 2.92) | 0.00e+00 | 2.79*** (2.68, 2.90) | 0.00e+00 | 2.78*** (2.66, 2.90) | 0.00e+00 | 2.68*** (2.57, 2.79) | 0.00e+00 |
| ^1^*p<0.05; **p<0.01; ***p<0.001; aOR = adjusted odds ratio; CI = confidence interval | | | | | | | | | | |
|  | | | | | | | | | | |
